# Design of MARQUIS2: study protocol for a mentored implementation study of an evidence-based toolkit to improve patient safety through medication reconciliation

**DOI:** 10.1186/s12913-019-4491-5

**Published:** 2019-09-11

**Authors:** Amanda S. Mixon, G. Randy Smith, Meghan Mallouk, Harry Reyes Nieva, Sunil Kripalani, Stephanie Rennke, Eugene Chu, Anirudh Sridharan, Anuj Dalal, Stephanie Mueller, Mark Williams, Tosha Wetterneck, Jason M. Stein, Deonni Stolldorf, Eric Howell, John Orav, Stephanie Labonville, Brian Levin, Catherine Yoon, Marcus Gresham, Jenna Goldstein, Sara Platt, Christopher Nyenpan, Jeffrey L. Schnipper, the MARQUIS2 Site Leaders, Sanchita Sen, Samer Badr, Michelle Murphy, Corrie Vasilopoulos, Tara Vlasimsky, Christine Roussel, Olugbenga Arole, Loredana Diana Berescu, Arif Arifuddowla, Hattie Main, Susan Pickle, Cristy Singleton, Brenda Asplund, Andrea Delrue, Andrea Forgione, Colleen Shipman, Luigi Brunetti, Hina Ahmed, Adrian Gonzales, Mithu Molla, Sarah Bojerek, Andrea Nguyen, Robert El-Kareh, Kyle Koenig, Loutfi Succari, Scott Kincaid, Pamela Proctor, Robert Pendleton, Amy Baughman, Kimberly Boothe, Katarzyna Szablowski, Olukemi Akande, Eric Tichy, the MARQUIS2 Study Group, Chi Zheng, Ryan Centafont, Regina Jahrstorfer, Lisa Jaser, Isha John, Margaret Curtin, Jenna Swindler, Joe Marcus, Robert Osten, Tian Yaw, Zainulabdeen Al-Jammali, Nancy Doherty, Brandi Hamilton, Magdee Hugais, Samson Lee, Paul Sabatini, Eddie Eabisa, Jennifer Mello, Julianna Burton, Edward Fink, Anthony Biondo, Trina Huynh, Ken Kormorny, Adonice Khoury, Kathryn Ruf, Dwayne Pierce, Chadrick Lowther, Karli Edholm, Shantel Mullin, Nicole Murphy, Jeni Norstrom, Laura Driscoll, Maribeth Cabie, Andrew Cadorette, Sara John, Amy D’Silva, Lionel Picot-Vierra

**Affiliations:** 1 GRECC, VA Tennessee Valley Healthcare System and Section of Hospital Medicine, Vanderbilt University Medical Center, Suite 450, 2525 West End Avenue, Nashville, TN 37203 USA; 2 Hospital Medicine, Northwestern Feinberg School of Medicine, Chicago, IL USA; 3 Center for Quality Improvement, Society of Hospital Medicine, Philadelphia, PA USA; 4 Division of General Medicine, Brigham and Women’s Hospital, Harvard Medical School, Boston, MA USA; 5 Section of Hospital Medicine, Vanderbilt University Medical Center, Nashville, TN USA; 6 Division of Hospital Medicine, University of California San Francisco Medical Center, San Francisco, CA USA; 7 Division of Hospital Medicine, Parkland Health and Hospital System and Department of Internal Medicine, University of Texas Southwestern School of Medicine, Dallas, TX USA; 8 Howard County General Hospital, Columbia, MD USA; 9 Department of Internal Medicine, University of Kentucky, Lexington, KY USA; 10 Division of General Internal Medicine, University of Wisconsin, Madison, WI USA; 11 1Unit, Atlanta, GA USA; 12 School of Nursing, Vanderbilt University, Nashville, TN USA; 13 Division of Collaborative Inpatient Medicine Service, Johns Hopkins Bayview Medical Center, Baltimore, MD USA; 14 Center for Hospital Innovation and Improvement, Society of Hospital Medicine, Philadelphia, PA USA

**Keywords:** Medication reconciliation, Patient safety, Hospital medicine, Transitions in care, Medication errors, Quality improvement

## Abstract

**Background:**

The first Multi-center Medication Reconciliation Quality Improvement Study (MARQUIS1) demonstrated that implementation of a medication reconciliation best practices toolkit decreased total unintentional medication discrepancies in five hospitals. We sought to implement the MARQUIS toolkit in more diverse hospitals, incorporating lessons learned from MARQUIS1.

**Methods:**

MARQUIS2 is a pragmatic, mentored implementation QI study which collected clinical and implementation outcomes. Sites implemented a revised toolkit, which included interventions from these domains: 1) best possible medication history (BPMH)-taking; 2) discharge medication reconciliation and patient/caregiver counseling; 3) identifying and defining clinician roles and responsibilities; 4) risk stratification; 5) health information technology improvements; 6) improved access to medication sources; 7) identification and correction of real-time discrepancies; and, 8) stakeholder engagement. Eight hospitalists mentored the sites via one site visit and monthly phone calls over the 18-month intervention period. Each site’s local QI team assessed opportunities to improve, implemented at least one of the 17 toolkit components, and accessed a variety of resources (e.g. implementation manual, webinars, and workshops). Outcomes to be assessed will include unintentional medication discrepancies per patient.

**Discussion:**

A mentored multi-center medication reconciliation QI initiative using a best practices toolkit was successfully implemented across 18 medical centers. The 18 participating sites varied in size, teaching status, location, and electronic health record (EHR) platform.

We introduce barriers to implementation and lessons learned from MARQUIS1, such as the importance of utilizing dedicated, trained medication history takers, simple EHR solutions, clarifying roles and responsibilities, and the input of patients and families when improving medication reconciliation.

## Background

### Problem description

Medication errors are a major patient safety concern during transitions in care and occur across all healthcare settings. Errors arise from medication discrepancies, defined as unexplained differences among documented regimens across different sites of care [1]. In prior work, we demonstrated that general medical inpatients experienced on average at least one discrepancy with potential for patient harm in either their admission or discharge medication orders [2]. When discrepancies are unintentional and unresolved, they can cause harmful adverse drug events (ADEs) and substantially increase health care costs [3, 4].

Medication reconciliation, defined as “a process of identifying the most accurate list of all medications a patient is taking … and using this list to provide correct medications for patients anywhere within the health care system,” is required at all care transitions to reduce actual and potential harm caused by medication discrepancies [5]. The Joint Commission (TJC) designated medication reconciliation as a National Patient Safety Goal (NPSG.03.06.01) in 2005, but it has proven difficult for institutions to implement [6, 7]. At many hospitals, the quality of medication reconciliation remains poor, and evidence-based practice is lacking [8].

### Available knowledge

To address this problem, we conducted the first Multi-center Medication Reconciliation Quality Improvement Study (MARQUIS1) at five US hospitals, with Agency for Healthcare Research and Quality funding [9]. This study consolidated medication reconciliation best practices and rigorously evaluated them in a real-world setting [10–12]. The multifaceted intervention, including a medication reconciliation toolkit along with mentored implementation, reduced total but not potentially harmful medication discrepancies in admission and discharge orders [13]. Implementation was variable across sites. Using mixed methods program evaluation and site-driven feedback, we identified the most important components of the intervention and many lessons for successful implementation. Furthermore, we refined the intervention toolkit and implementation guide and developed instructional videos, didactic presentations, and simulation exercises. This revised toolkit plus mentored implementation were then ready for use in a larger number of institutions to improve medication safety more broadly.

### Rationale

MARQUIS2 built upon the MARQUIS1 project, which predominantly relied on the Brown and Lilford patient safety intervention framework of clinical process-oriented interventions complemented by management-oriented interventions [14–17]. Our rationale of the current study was: If hospitals were to adopt the MARQUIS2 toolkit, then medication discrepancies would be reduced, so that patient safety at care transitions would be improved.

The specific aims of MARQUIS2 were to:

- Implement the refined MARQUIS2 evidence-based medication reconciliation toolkit at 18 diverse hospitals, using a mentored quality improvement (QI) implementation model.
- Evaluate the effect of the MARQUIS2 program on unintentional medication discrepancies.
- Inform future spread of medication reconciliation interventions by performing an evaluation of program implementation using the Reach, Effectiveness, Adoption, Implementation, Maintenance (RE-AIM) framework [18].

This manuscript describes the design of MARQUIS2, informed by lessons learned from MARQUIS1.

## Methods

### Context and sites

We identified potentially interested hospitals from: MARQUIS1 toolkit downloads from the Society of Hospital Medicine (SHM) website; attendance at prior MARQUIS1 workshops or other MARQUIS talks; participation in other SHM mentored implementation projects; and emails sent to SHM’s members and prospect list. Interested sites completed an application, which asked sites to identify a site leader and QI team, the hospital’s characteristics and institutional environment, experience with prior QI projects and specific medication reconciliation efforts, a needs assessment, and their goals, strengths, and areas for improvement. Each application was reviewed by two mentors and presented to the study team. Applications were rated using the following criteria: 1) strong institutional support, demonstrated by a letter from an executive champion; 2) a local site leader with QI knowledge, experience, and dedicated project time; 3) an interdisciplinary QI team with appropriate roles delineated; 4) institutional experience with successful patient safety and QI projects; 5) local financial support and resources to collect data, including a study pharmacist(s) with dedicated time; and, 6) intention to implement one or more intervention components.

**Fig. 1:**
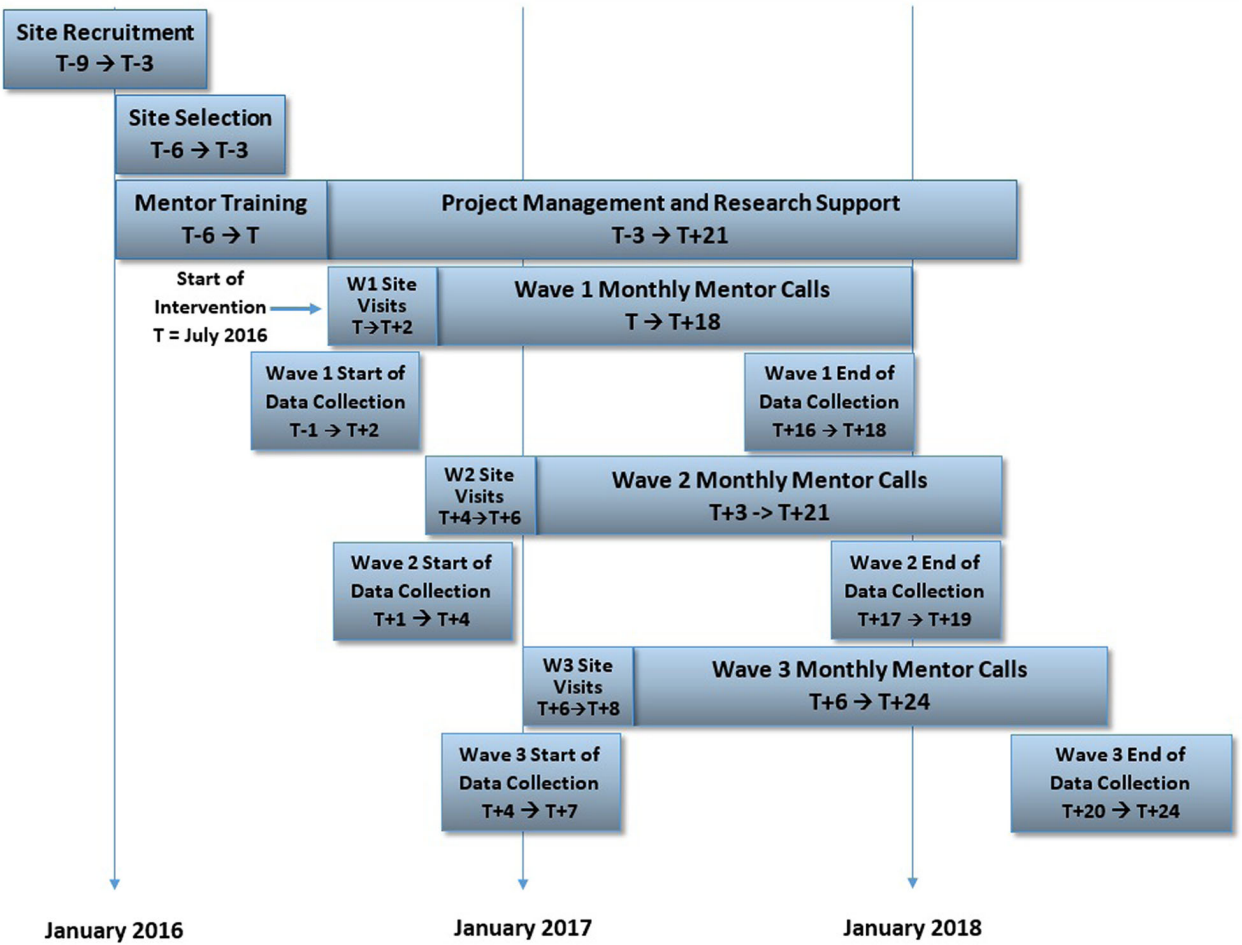
Study timeline

From 72 applications we selected 18 sites, chosen purposefully for heterogeneity in characteristics such as size and location. We excluded sites which had already implemented 2 or more interventions from the MARQUIS1 toolkit. Sites were divided into three waves of staggered implementation based on their planned implementation timeline, with each implementation wave including 6 sites and lasting 18 months (Fig. 1). The first sites started mentored implementation in April 2016, and the last sites ended mentored implementation in April 2018.

### Study design

To achieve both the research and quality improvement aims of MARQUIS2, data were collected in real time during the intervention. Site teams entered data into Research Electronic Data Capture (REDCap), a secure web-based platform for data management [19]. The data coordinating center staff were tasked with analyzing and feeding back site-specific data to each site in real time. Thus, data collection was achieved for research purposes while sites were concurrently able to utilize their data for ongoing refinement of their local QI initiatives.

### Mentored implementation

Each site team included interprofessional hospital-based members and was generally led by physician and pharmacist co-leaders, although this varied by site. The site team was responsible for the MARQUIS2 goals with guidance from a mentor. All eight mentors were hospitalists recruited from several academic institutions with experience in QI methods, mentoring healthcare professionals, and/or medication safety. They were assigned up to three sites each. Mentors underwent an all-day orientation to the MARQUIS2 project and “Mentor University” training at the SHM’s national office at the start of the study [20, 21]. Senior mentors, who had experience as implementation mentors for MARQUIS1, provided ongoing guidance to mentors and attended each mentor’s first site visit and first two monthly site calls with them. Mentors also engaged in monthly “mentor council” calls with the principal investigator and senior mentors to report on site progress, discuss challenges encountered, seek advice, and share activities associated with successful implementation.

Based on prior mentored implementation projects, monthly site calls and at least one site visit are critical for a site’s success [21]. The site team and assigned implementation mentor engaged in at least an hour-long conference call each month for the 18-month implementation period, which focused on reviewing data collection/analysis and facilitating toolkit implementation [20, 21]. Mentors also engaged in a two-day site visit within the first 6 months of the mentoring relationship, specifically chosen early in the implementation period due to lessons learned from MARQUIS1 regarding the value of conducting site visits as early as possible. During the site visit, the mentor directly observed the current medication reconciliation practices, strengthened relationships with the site team and site executive leadership, discussed possible interventions, and addressed barriers to MARQUIS2 toolkit implementation. This direct contact between sites and mentors early in the implementation period allowed for easier identification of barriers to implementation and garnered executive leadership and other stakeholder support, facilitating successful participation in the study.

### Interventions

#### Differences between MARQUIS 1 and 2

Table 1 denotes the differences grouped by changes to the toolkit, the implementation approach, and the analysis plan. (See relevant sub-sections below.)

**Table 1:** Differences between MARQUIS1 and MARQUIS2

Domain	Specific aspect	MARQUIS1	MARQUIS2
Site Selection	How and when sites were recruited	Informal process, sites identified prior to submission of grant application	Widespread search, formal application process, most sites identified at beginning of study period
Toolkit	Best possible medication history (BPMH) training	Didactic materials only, including slide presentations and videos	Didactic materials plus simulation materials with standardized cases and role-playing to enhance learning and verify competency
	Role of staff taking BPMH	Agnostic to type of personnel	Increased emphasis on the value of pharmacy technicians as “medication reconciliation assistants” trained to take accurate medication histories
	Return on investment	Rudimentary calculations	More precise calculations based on MARQUIS1 data
	Patient counseling tools	Didactic materials, including slide presentations and videos	Enhanced didactic materials plus scripts and worksheets developed with Patient and Family Advisory Council (PFAC) input
Implementation Approach	Site team training	Webinars	Webinars + 4 regional workshops
	Site visits, number and timing	2 site visits: first visit in months 5–10, second in months 16–19	1 site visit within first 6 months
	Patient-family engagement	No formalized program	Established and engaged PFAC in monthly discussions
	Inter-site sharing	No formalized sharing	3 peer-to-peer webinars featuring sites’ stories of successes and challenges
	Health information technology (HIT)	Discovered significant challenges exist with the design, implementation, and use of HIT during medication reconciliation processes that, together with health systems issues, impacted medication safety	Provided guidance on how best to work with existing HIT—e.g. allowing pharmacists to make changes to medication lists, documenting the quality of the medication history taken and its sources, and customizing discharge instructions to make medication changes clear to patients and clinicians
Analyses	Intervention assessment	Scoring system of interventions; categorization of site-level intervention components based on meeting minutes analyzed retrospectively; no data on receipt of interventions at the patient level	Prospective collection of site-level interventions based on monthly site surveys; prospective collection of patient-level interventions as part of data collection on discrepancies
	Outcome assessment	Total medication discrepancies with potential for harm, involving adjudication; total medication discrepancies	Total medication discrepancies per medication per patient, as adopted by the Leapfrog Group [22]
	Program evaluation	Surveys, direct observation, interviews, focus groups of contextual factors, intervention fidelity	RE-AIM^a^ framework

^a^Reach, Effectiveness, Adoption, Implementation, and Maintenance

#### Site team training

All 18 sites participated in educational webinars at the beginning of their respective implementation wave. Site teams participated in subsequent educational webinars on the MARQUIS2 toolkit, research data collection responsibilities, and techniques to train other healthcare clinicians on medication reconciliation best practices. Webinars were recorded for those sites unable to attend the live events. Additionally, SHM coordinated four optional regional workshops (New York, New Jersey, Pennsylvania, and California) for sites to learn best practices in person; 14 sites attended these in-person events. These workshops covered: generating institutional support; training others to take a BPMH and giving them feedback; training on discharge medication counseling; and, identifying and overcoming barriers to implementation, sustainment, and spread. During the workshops, site leaders could network with other site leaders and share their experiences.

Each site identified at least one study pharmacist responsible for data collection on medication discrepancies (see Outcome Assessment). The site pharmacists were trained by the central study pharmacist using standardized patient cases during two online webinar sessions. In addition, throughout the intervention period, SHM and the data coordinating center conducted training sessions for site personnel on how to measure medication discrepancies in a consistent way across sites over time.

#### Toolkit

The MARQUIS2 toolkit consists of 17 interventions to improve medication reconciliation, grouped into eight domains: taking the best possible medication history (BPMH); discharge medication reconciliation and counseling; clarifying roles and responsibilities; risk stratification; health information technology improvements to the electronic health record (EHR); improving access to medication sources; “measure-vention”, i.e. measuring then intervening to correct discrepancies in real time; and, stakeholder engagement [23]. All sites were provided an updated implementation manual, instructional videos, presentations, and return-on-investment calculators (i.e., from investing in medication reconciliation personnel).

The toolkit was upgraded with changes reflecting our experience from implementation of MARQUIS1 (Table 1) [10]. First, we developed simulated standardized cases for taking BPMHs and certifying competency, since the receipt of didactic education in BPMH-taking did not always correlate with mastery of skills in MARQUIS1. Second, we emphasized the value of pharmacy technicians as “medication reconciliation assistants” trained to take accurate medication histories [24]. Adoption of such a program was associated with significant reduction in total discrepancies at one site and spread from that site to several others during MARQUIS1 [13]. We also learned from MARQUIS1 that medication histories are best taken as early in the hospitalization as possible, ideally before admission orders are written, to avoid re-work when errors are corrected. Third, we developed more precise return-on-investment (ROI) calculations based on MARQUIS1 study results and the literature on the impact of harmful discrepancies. We learned that accurate ROI calculations are critical when gaining buy-in from and obtaining budget approval for staffing changes by local hospital leadership. Fourth, MARQUIS1 implementation was adversely affected by several sites concurrently implementing new vendor EHRs. At the time of MARQUIS2 most sites already had established vendor EHRs, so we focused on relatively simple changes they could make to their existing systems, including standardized note templates for medication history-takers and giving proper permissions to personnel to make medication history changes in the EHR. Lastly, we increased our emphasis on the patient-centered components of the intervention (e.g., patient and caregiver counseling at discharge), with the development of tools such as clinician scripts and worksheets. These tools were iteratively refined by the Patient and Family Advisory Council (PFAC) early in the study.

### Patient and family advisory council (PFAC)

With growing evidence of the value of input from patients and their caregivers in systems improvement research [25, 26], we assembled a PFAC for MARQUIS2. The PFAC consisted of a PFAC specialist who had worked on prior projects with the principal investigator and four additional members recruited from mentor and/or co-investigator institutions. Members were chosen based on relevant experience as a patient or caregiver (e.g., having suffered an adverse drug event during transitions of care) and their ability to work collaboratively and advocate for other patients and families. The PFAC participated in monthly phone meetings and engaged in every aspect of the study, including the design of patient-facing interventions (such as social marketing campaigns aimed at patients), overall study design, data collection, and interpretation, dissemination, and implications of the study’s results. During the monthly calls, we updated the PFAC on study progress and asked them to provide feedback on results and guidance on needed changes*.*

### Project management

SHM’s Center for Quality Improvement served as the project management office, driving timelines and program milestones for each site on a day-to-day basis. SHM marketed the study, recruited sites to participate, supported the evaluation and selection of applicants, and trained program mentors at Mentor University [20, 21].

Once sites and mentors were selected, most project management activities were managed through QuesGen (QuesGen Systems, Burlingame, CA), an online platform accessible to site teams, mentors, and SHM. Specifically, the website housed minutes from mentor-mentee monthly phone calls, action items, data tables and charts displaying ongoing process and outcome data, and anonymized benchmarking data for each wave. Furthermore, SHM provided dashboards displaying the site’s month-to-month progress towards key milestones using stoplight color coding to indicate whether the site had completed, begun working on, or were behind on milestones.

SHM staff scheduled all recurrent calls between mentors and their sites as well as monthly “mentor council” calls. SHM project managers coordinated all aspects of the site visits. Lastly, SHM hosted quarterly peer-to-peer webinars for sites to present their work, share their successes and challenges, and learn from each other.

### Study of the intervention

Sites were engaged for 18 months of intervention time. During this period, sites were responsible for collecting data, implementing interventions, submitting monthly intervention tracking data, participating in study training sessions for site personnel on collecting consistent data, participating in monthly mentor calls, and hosting a site visit from their mentor.

### Outcome assessment

The primary research outcome was the total number of unintentional medication discrepancies in admission and discharge orders per patient, similar to MARQUIS1 [9]. Each site was commissioned to obtain a gold standard medication history on a random sample of 22 patients per month [27]. This sample included both control patients and intervention patients. Intervention patients were defined as those who received a patient-level intervention (e.g. BPMH was taken in the ED by a dedicated, MARQUIS-trained clinician) and/or a system-level intervention (e.g. the site clarified and assigned roles and responsibilities to different staff regarding the medication reconciliation process) implemented on the unit where the patient was admitted. Patients were admitted to one of several pre-specified adult, non-critical care medical or surgical unit(s) and hospitalized long enough to obtain a “gold standard” medication history. The study pharmacist gathered the gold standard medication history, separate from and in addition to any medication reconciliation activities completed by healthcare clinicians for patient care. The study pharmacist’s gold standard medication history was then compared to the clinician-generated medication history, the admission orders, and the discharge orders for each patient. Unintentional discrepancies in orders were identified as previously described and recorded in REDCap [9, 19]. The pharmacist also recorded the timing of the discrepancy (admission vs. discharge), type (omission, additional medication, change in dose, route, frequency, or formulation), and reason (history error vs. reconciliation error) [2]. Lastly, if applicable, they documented which patient-level interventions each patient received.

To give ongoing feedback to sites regarding the effect of intervention(s) on outcomes, we provided monthly data on discrepancy rates using an on-treatment analysis of patient- and system-level interventions. De-identified discrepancy data were extracted from REDCap by the data coordinating center for this feedback. Site QI teams were also able to access monthly reports of their progress to reduce medication discrepancies over time across all study units, in the form of statistical process control charts.

In MARQUIS2, physicians did not adjudicate medication discrepancies for potential for patient harm, as this process is labor intensive and expensive. The elimination of adjudication and the collection and merging of patient-level administrative data from each site greatly reduced the study costs and data collection burden, allowing us to enroll 18 sites for the same budget as 5 sites in MARQUIS1 (Table 1). Moreover, from our prior studies we found that improvements in potentially harmful medication discrepancies have a similar direction and magnitude as improvements in total discrepancies [28].

Sites also tracked which system-level interventions they implemented by completing a brief monthly survey. Through this prospective data collection of site-level interventions implemented, we improved upon the process used for MARQUIS1, in which we had retrospectively gathered this information from monthly mentor call minutes (Table 1). Mentors additionally encouraged sites to collect local relevant process and outcome measures, such as tracking the workload of pharmacists involved in discharge counseling.

### Evaluation of implementation

One of the specific aims of MARQUIS2 was the evaluation of the program using the Reach, Effectiveness, Adoption, Implementation, and Maintenance (RE-AIM) framework [18]. Having already conducted more intensive mixed methods analyses in MARQUIS1, RE-AIM allowed us to focus more on widespread implementation and maintenance (Table 1). Table 2 frames the definitions and data sources to measure the RE-AIM components, which include: characteristics of hospitals that did and did not apply; descriptive statistics of units and services that adopted and did not adopt the intervention at each site; unintentional medication discrepancies in admission and discharge orders; monthly survey data of system-level interventions in use; proportion of patients that received patient-level interventions; and, measurement of discrepancies 6 months after the intervention period. The sustainability assessments were developed specifically for MARQUIS2 based upon existing literature [29–33].

**Table 2:** RE-AIM measures

Dimension	Definition	Application to study
Reach	Absolute number, proportion, and representativeness of individuals willing to participate in a given initiative	Number and characteristics of sites that participated, applied but were not selected to participate, sites that were contacted but did not apply, and all US hospitals; Number and characteristics of stakeholders within each institution involved in the study at each participating site
Effectiveness	Impact of intervention on outcomes	Primary outcome: number of unintentional medication discrepancies in admission and discharge orders per patient
Adoption	Absolute number, proportion, and representativeness of settings and agents willing to initiate the program	Description of services and units that did and did not adopt the intervention
Implementation	Fidelity to the various elements of an intervention’s protocol	Number and type of system-level intervention components implemented; proportion of patients that received patient-level interventions
Maintenance	Extent to which a program becomes part of routine practice; long-term effects of a program on outcomes	Discrepancy rates 6 months after the end of mentored implementation; intervention components still in place and participating units/services 6 months after the end of mentored implementation.

### Planned statistical analyses

This protocol does not include reporting of outcomes. However, we plan to use interrupted time series methodology and a random effects analysis on all patients evaluated for discrepancies across the 18 sites, similar to those conducted for MARQUIS1 [9]. We will analyze the primary outcome of total number of unintentional medication discrepancies in admission and discharge orders per patient using a Poisson regression model, with the number of preadmission medications as a model offset. Random effects will be included in the regression model to account for clustering at the level of the site (due to common practice styles or institutional culture). Temporal trends, or improvements over time, will be incorporated into the regression model as slopes and treated as random effects in order to allow each site to have a unique trend prior to the introduction of any interventions. In this way, each site will serve as its own control. The main outcomes of interest will be changes in y-intercept (sudden improvements with introduction of the intervention) and changes in slope (changes in temporal trends after implementation begins). Improvements in discrepancies over time will be the result of several factors: the effectiveness of individual intervention components (including their iterative refinement), the number of components implemented, the fidelity with which each intervention is implemented, and the spread and sustainability of implementation on participating services and units. We plan to adjust for a limited number of fixed, patient-level covariates based on the prior literature and MARQUIS1: patient age, medical versus surgical service, patient understanding of their medications (determined by the study pharmacist using standardized criteria [2]), and season.

To determine the effects of various intervention components, we will conduct a secondary analysis similar to that in MARQUIS1 [9]. First, we will categorize all system-level interventions conducted by any site by component, including date(s) of implementation or substantial spread (to a new group of patients or clinicians), based on the monthly site surveys. This approach is less burdensome to site leaders than the scoring system used in MARQUIS1. We will then analyze the data using Poisson regression to detect sudden reductions in unintentional medication discrepancy rates temporally associated with each implementation or spread of each intervention component across all sites. Additionally, we will look at the effects of each patient-level intervention on patients who received them versus those who did not.

### Power and sample size

We recommended that each site collect data on approximately 22 patients per month. Across all 18 sites, this will total 7128 patients. Based on prior experience we expected that it would take an average of 6 months of mentorship for sites to begin implementing interventions, which meant 12 months of intervention delivery in the 18 month period of mentored implementation. Thus we expected to have data on approximately 2376 patients pre- and 4752 patients post-intervention. Power calculations are based on our MARQUIS1 data which showed that the (Poisson distributed) number of unintentional medication discrepancies was reduced by 11% from an average of 2.9 per patient to 2.58 per patient. In MARQUIS2 we will have over 99% power to detect a comparable overall effect. The study will also be powered to examine the effect of individual intervention components. Even if an intervention component is only introduced at a single site, we will have data on 150 patients pre-intervention and 300 patients post-intervention, yielding 90% power to detect a reduction in discrepancies from 2.9 per patient to 2.4 per patient. Finally, we will have 80% power to detect a difference between an 11% relative reduction in MARQUIS1 and a 24% reduction in the current study.

## Discussion

Figure 1 displays the study timeline. The beginning of the study was characterized by site recruitment, selection, and mentor training. The next phase was baseline data collection by the three waves of sites, followed by site visits and monthly mentor calls, which lasted 18 months for each wave.

The site characteristics are listed in Table 3. The 18 sites varied in hospital size, location, urbanization, academic affiliation, and EHR. Hospital size ranged from 88 to 1551 beds. With the exception of the Southwest, all U.S. regions are represented, plus one site in Canada. Types of hospitals and health systems ranged from publicly funded safety net hospitals, to community hospitals, to university medical centers as well as one Department of Veterans Affairs facility. The EHRs represent those with the largest market share nationally.

**Table 3:** Characteristics of participating sites

Site	# Beds	Region	Location (Urban, Suburban, Rural)	Teaching Status	Profit Status	EHR
A	534	Northeast	Urban	University Medical Center	Non-profit	Epic
B	88/160	Northeast	Rural	Community Teaching	Non-profit	Meditech
C	266	Northeast	Suburban	Community Hospital with Some Teaching Opportunities	Non-profit	Epic
D	255	West	Urban	Community Teaching	Non-profit	Cerner
E	563	West	Suburban	University Medical Center	Non-profit	Epic
F	638	West	Urban	University Medical Center	Non-profit	Epic
G	453	South	Suburban	Community Teaching	Non-profit	Cerner Soarian
H	836^a^	Ontario, Canada	Suburban/77% Large Urban, 11% Small pop. Centre, and 12% Rural	Community Teaching/multi-site community hospital, partnership with McMaster Medical school and accept learners	$500 M Budget	None
I	576	West	Urban	University Medical Center	Non-profit	Epic
J	365	Northeast	Suburban	Community Teaching	Non-profit	Cerner/Allscripts
K	627	West	Urban	University Medical Center	Non-profit	Epic
L	1541	Northeast	Urban	University Medical Center	Non-profit	Epic
M	525	West	Urban	County - Publicly-funded safety net hospital	Non-profit	Epic
N	232	Northeast	Suburban	Community Non-teaching	Non-profit	Meditech
O	763	South	Urban	Community Teaching	Non-profit	Cerner - Intermed RxHX
P	850/996	South	Suburban	University Medical Center	Non-profit	Epic
Q	744	South	Urban	University Medical Center	Non-profit	Allscripts
R	112^b^	Northeast	Suburban	Department of Veterans Affairs	Non-profit	Computerized Patient Record System (CPRS)

^a^456 Acute Beds, 150 Mental Health Beds, 115 Long-Term Care Beds, 177 Complex Care Beds

^b^65 Sub-acute Beds, 32 Long-term Care Beds, 15 Hospice and Palliative Care Beds

In summary, we recruited 18 diverse hospitals to participate in the MARQUIS2 mentored implementation study. In this large pragmatic quality improvement study, we implemented lessons learned from mixed methods QI studies such as MARQUIS1 on how to improve interventions and the approach. We streamlined the MARQUIS2 toolkit and placed emphasis on interventions shown to reduce harmful medication discrepancies: utilizing dedicated medication history takers, training existing staff to perform discharge medication reconciliation and patient counseling, and clarifying roles and responsibilities among clinical personnel [34]. We also disseminated solutions to known HIT challenges and cautioned sites on implementing new EHRs. We incorporated a PFAC into all steps of the study, integrating patient and caregiver stakeholders into all phases of the research. Lastly, we improved the efficiency of data collection. If the MARQUIS2 interventions are successful in reducing unintentional medication discrepancies, we plan to disseminate widely our findings to improve medication safety across an even wider range of health systems.

## Data Availability

The protocol, statistical code, and de-identified and anonymized dataset are available from Dr. Schnipper with a reasonable written request.

## Abbreviations

ADEs: Adverse drug events
BPMH: Best possible medication history
EHR: Electronic health record
HIT: Health information technology
IRB: Institutional Review Board
MARQUIS1: Multi-center Medication Reconciliation Quality Improvement Study
MARQUIS2: Multi-center Medication Reconciliation Quality Improvement Study 2
NPSG: National Patient Safety Goal
PFAC: Patient and family advisory council
QI: Quality improvement
RE-AIM: Reach, Effectiveness, Adoption, Implementation, Maintenance
REDCap: Research Electronic Data Capture
ROI: Return-on-investment
SHM: Society of Hospital Medicine
TJC: The Joint Commission
VA: Department of Veterans Affairs
